# Investigation of a Novel Hepatitis B Virus Surface Antigen (HBsAg) Escape Mutant Affecting Immunogenicity

**DOI:** 10.1371/journal.pone.0167871

**Published:** 2017-01-03

**Authors:** Md. Golzar Hossain, Keiji Ueda

**Affiliations:** Division of Virology, Department of Microbiology and Immunology, Osaka University Graduate School of Medicine, Suita, Osaka, Japan; Centre de Recherche en Cancerologie de Lyon, FRANCE

## Abstract

Mutation in the hepatitis B virus surface antigen (HBsAg) may affect the efficiency of diagnostic immunoassays or success of vaccinations using HBsAg. Thus, antigenicity and immunogenicity analyses of the mutated HBsAg are necessary to develop novel diagnostic tools and efficient vaccinations. Here, the *in vitro* antigenicity of three wild-type HBsAg open reading frames (ORFs) (adr4, W1S [subtype adr] and W3S [subtype adr]) isolated from clinically infected patients and nineteen synthesized single/double/multiple amino acid-substituted mutants were tested with commercial ELISA kits. Immunofluorescence staining of transfected cells and Western blot analysis confirmed that these ORFs were expressed at comparable levels in HEK-293 cells. W1S and adr4 were clearly detected, whereas W3S could not be detected. Using the same commercial immunoassay kit, we found that the single mutants, K120P and D123T, were marginally reactive, whereas W3S-aW1S and the double mutant, K120P/D123T, exhibited antigenicity roughly equivalent to the wild-type wako1S. On the other hand, the single mutants of W1S, P120K and T123D, significantly impaired the reactivity, while W1S-aW3S and the double mutant of W1S, P120K/T123D, resulted in a complete loss of antigenicity. In addition, ELISA revealed reduced HBs antigenicity of two mutants, W1S N146G and W1S Q129R/G145R. These commercial ELISA-based antigenic reactivities of HBsAg were also strongly correlated with the predicted A*i* alterations of affected amino acids due to the specific mutation. In conclusion, this study showed for the first time that lysine (K120) and aspartate (D123) simultaneously affected HBsAg antigenicity, leading to diagnostic failure. These findings will improve diagnostic assays and vaccine development.

## Introduction

More than 350 million people worldwide are chronically infected with hepatitis B virus (HBV), which is considered one of the major human pathogens causing hepatitis, liver cirrhosis and hepatocellular carcinoma (HCC) [1, 2]. HBV is a small DNA virus belonging to the Hepadnaviridae family. It is around 42 nm in diameter with an icosahedral nucleocapsid. The HBV genome contains only 3.2 kb of partially double-stranded circular DNA, which includes four alternative overlapping open reading frames (ORFs) and replicates through an RNA intermediate [3, 4]. The viral genome is then transported into the host cell nucleus to form a complete and covalently closed circular DNA (cccDNA). By using the host cellular machineries, the cccDNA generally synthesizes four kinds of viral mRNAs. These are the 3.5 kb precore mRNA and pregenomic RNA (pgRNA), 2.4 kb and 2.1 kb surface antigen mRNAs and 0.7 kb X protein mRNA [5]. The complete infectious virions, also known as Dane particles, are enveloped by three virus-coded surface (S) proteins-namely, the large, middle and small S proteins (LS, MS and SS, respectively) [6, 7]. These HBV surface proteins are all related to each other by sharing a common C-terminal S domain (SS) as they contain each start site with a common termination codon.

The small surface HBs (SS) is the prime component of the enveloped viral particles and is expressed and secreted in greater excess than LS or MS on the mature virus particles. The hepatitis B virus small surface antigen (HBsAg) is composed of 226 amino acids (aa) and the region from aa 99 to aa 169 is called a major hydrophilic region (MHR) [8]. There used to be four subtypes of HBsAg (adw, ayw, adr and ayr), defined by a common “a” determinant and two mutually exclusive determinants pairs, d/y and w/r. The determinant d has a lysine at residue 122, while y has an arginine; similarly w has a lysine at residue 160, while r has an arginine [9, 10]. It has been reported that the “a” determinant is located on one of the extra-membranous loop spanning amino acid residues 101 to 159 within the MHR [11]. In addition, the HBsAg is posttranslationally modified by N-linked glycosylation at aa 146 and produces two isoforms, glycosylated (gp27) and non-glycosylated (p24) [12].

The HBsAg is a hallmark of HBV infection, and as the first serological marker to appear during acute HBV infection, HBsAg is critical to diagnosis [13]. In addition, a recombinant HBsAg produced in yeast is used as a vaccine to prevent HBV infection [14, 15]. The MHR of HBsAg contains a highly conformational B-cells epitope cluster that is the main target of neutralizing antibodies to HBsAg [16, 17]. The high mutation rate due to lack of proofreading activity of HBV polymerase causes frequent mutations in the S domain of HBsAg, as it is overlapped completely by the polymerase gene [3, 18]. Mutation in the MHR alters the antigenicity and the antibody-binding specificity and leads to a failure of HBV detection (diagnostic escape) by conventional routine diagnostic HBsAg assays [17, 19–21]. Molecular analysis of viral samples collected from chronically infected patients has revealed that HBV DNA could be detected in blood sera and liver tissues with negative HBsAg [22–24]. Moreover, the HBsAg expression level may also be regulated and reduced by undescribed posttranslational mechanisms, and mutations in the HBsAg sequence could also be the cause of the decrease in HBsAg expression and secretion efficiency [25–27]. Reduced expression, deficient secretion into the extracellular space and inefficient binding capacity of HBsAg in commercial immunoassays could in turn lead to the frequent detection failure observed in individuals with occult hepatitis B virus infection (OBI) [19, 28]. The first vaccine-induced natural mutation (G145R) was found in a newborn of an HBsAg carrier mother, and mutations in this surface protein have also been shown to lead to a failure of immune prophylaxis in infants receiving HBV vaccines (“immune escape”) and in a liver transplant patient receiving hepatitis B immune globulin (HBIG) [29–31]. In addition, recent epidemiological, molecular and experimental studies have shown that such vaccine-escape HBsAg mutants should be circulating in pre-immunized individuals [32–36]. Because of these diagnostic and vaccination failures, infected individuals will become chronic silent carriers due to presence of the virus in the hepatocytes and the peripheral blood unless new diagnostic assays and vaccine strategies are developed [33, 37, 38]. Although there have been some reports on hepatitis B antigenicity and mutational analyses that were carried out either by site directed mutagenesis or isolated from patients, the phenotypic conformation and the antigenic properties of HBsAg remain obscure due to the continuous incidence of natural mutations [17, 19, 39]. Therefore, in order to develop novel diagnostic and vaccine strategies for hepatitis B, it will be necessary to examine the amino acid sequence of HBsAg in greater detail, since mutations in this sequence affect both the antigenicity and immunogenicity of HBsAg [28, 40–43].

In this study, we cloned the ORFs of three wild-type HBsAgs, designated W1S, W3S and adr4 (all of which were isolated from HBV-infected patients) and constructed a total of 19 synthetic mutants into a mammalian expression vector. Then, we checked and analyzed the antigenicity of expressed HBsAg in mammalian cells with commercial serological assays (based on enzyme-linked immunosorbent assay; ELISA). The expression of HBsAgs in HEK293-T cells was confirmed by immunofluorescence assay (IFA), Western blotting analysis and ELISA on nickel-coated plates with a mouse monoclonal anti-tag antibody. W1S but not W3S was detected with commercial ELISA kits. Mutational analyses revealed that double mutations with lysine at amino acid position 120 (120K) and aspartate at position 123 (123D) profoundly affected the antigenicity of HBsAg, possibly by changing its conformation, and led to a diagnostic failure of HBV infection. Single amino acid mutation of either P120K or T123D, or other positions such as 145 (G145R) or 146 (N146G), only marginally reduced the binding efficiency of HBsAg. Thus, double mutation of P120K and T123D will be crucial for the future design of hepatitis B vaccines and diagnostic assays.

## Materials and Methods

### HBsAg clones

The HBs clones used in this study were all patient-derived. HBV adr4 was a cloned HBV DNA (GenBank accession No. X01587). Two other HBs, W1S and W3S cDNA, were cloned from sera used as the panel sera to check the performance of diagnostic machines for HBV infection. They were very old samples and we could not specify the patients. The HBs ORF was amplified by PCR using the primers 5’-AAGGATCCCATGGAGAACACAACATCAGG-3’ and 5’-AACTCGAGTTAAATGTATACCCAAAGACA-3’. The amplified fragment was purified and digested with BamHI + XhoI, then cloned into an expression vector (see below) and sequenced. Mutant DNA clones of the HBs ORF were synthesized by Gene Art (Life Technologies) and cloned into the same expression vector.

### Plasmids

A mammalian expression vector, pEBVHis (Invitrogen), was used for expression. The ORF encoding HBsAg was cloned into the pEBVHisA or B vector. All wild-type (wt) and mutant (mt) HBsAgs with these vectors carried an N-terminal polyhistidine (His 6-tag) and an Xpress epitope (DLYDDDDK), which allowed the detection of wt- and mt HBsAg by Western blot and immunofluorescence assay (IFA) using a monoclonal antibody (mAb) against the His tag or the express epitope, since some wt- and mt-HBsAgs might not be properly detected by anti-HBs antibodies [39]. The his tag was also used for the immobilized metal affinity chromatography purification of recombinant HBsAg and his-tag binding assay with a nickel (Ni)-coated plate (Sigma HIS Select).

### Cell lines and transfection

HEK293-T cells, which was originally obtained from ATCC and maintained in our laboratory, were used for the expression and the detection of all wt- and mt-HBsAg. The cells were maintained in Dulbecco’s modified Eagle’s medium (Nacalai Tesque) supplemented with 10% fetal bovine serum (Equitech-Bio Inc.), 100 U/mL penicillin G, 100 μg/mL streptomycin, and 0.25 μg/mL amphotericin B at 37°C in an atmosphere of 5% CO_2_. Transient transfection was performed with 10 μg plasmid DNA in 1×10^6^ cells/100 mm dish overnight by using TransIT-LT1 reagent (Mirus^™^) according to the manufacturer’s guidelines. After 72 h, the supernatant was harvested and the cell lysate was prepared for detection of the expressed HBsAg by ELISA and for Western blot analysis. For IFA staining, transfection was performed in an 8-well tissue culture chamber slide (Matsunami Glass, Japan) with 400 ng plasmid/well (0.75×10^4^ cells/well) and incubated for 72 h using the same procedure.

To detect expressed HBsAg, the transfected cells and the culture supernatants were collected. The culture supernatant was passed through a 0.45 μm filter (Millex) to remove the cellular debris and subjected to ELISA (HBs S Antigen Quantitative ELISA Kit, Rapid II; Beacle Inc., Japan) or to polyethylene glycol (PEG) precipitation or purification for Western blotting analysis. The adherent monolayer of the cells on the dishes was washed two times with phosphate-buffered saline (PBS), harvested from the dishes with a cell scraper and collected in sterile 15 mL tubes by centrifugation at 250 × g for 5 min at 4°C. The cell pellet was then lysed in nickel NTA buffer A (50 mM NaH_2_PO_4_ [pH: 8], 300 mM NaCl, 0.1% NP40 and a complete protease inhibitor for His-tagged protein [1:1000] [Sigma P8849]). The cell debris was removed by centrifugation at 8400 × g at 4°C for 30 min and the collected lysates were used for ELISA or His 6-tagged protein-binding assay and for purification of HBsAg to detect by Western blot. The targeted proteins from the cell lysates and the culture supernatant were then purified by immobilized metal affinity chromatography. Briefly, the cell lysates and the culture supernatant were incubated overnight at 4°C with rotation after adding 100 μl Ni-NTA agarose beads (His-Select; Sigma) to nickel NTA buffer A supplemented with 10 mM imidazole. The agarose beads were then washed three times with nickel NTA buffer B (50 mM NaH_2_PO_4_ [pH: 8.0], 300 mM NaCl, 20 mM imidazole and 0.05% Tween 20). The bound protein was eluted with nickel NTA buffer C (50 mM NaH_2_PO_4_ [pH: 8.0], 300 mM NaCl, 250 mM imidazole and 0.05% Tween 20). These eluted fractions were used as samples for Western blotting to detect wt- and mt-HBsAg.

For PEG precipitation, 30% (w/v) PEG8000 (Wako Pure Chemical Industries, Ltd.) solution was added to the culture supernatants at a final concentration of 6% and incubated at 4°C overnight after mixing. The samples were then centrifuged at 8400 × g for 30 min at 4°C and the supernatant was discarded completely. The pellet was dissolved in PBS and subjected to identification by his 6-tagged protein-binding assay with a Ni-coated plate.

### His 6-tagged protein-binding assay (reactivity of HBsAg with anti-tag Ab [%])

The Ni-coated plate (HIS-Select High Capacitty (HC) Nickel coated plates; Sigma-Aldrich) was blocked with Super Block (Pierce), then dried and kept at 4°C until use. The PEG-precipitated culture supernatants or the cell lysates were loaded onto the 96-well Nickel-coated plate and incubated at 37°C for 1 h followed by washing three times with TBS-T (20 mM Tris-HCl pH 8.0, 150 mM NaCl, 0.1% Tween 20; Tris-buffered saline containing 0.1% Tween 20). The plate was then incubated for 30 min at 37°C with a mouse mAb (anti-Xpress^™^ antibody; Invitrogen) and again washed three times with TBS-T. The plate was reacted with anti-mouse IgG conjugated with horseradish peroxidase (HRP) (Dako) for 30 min at 37°C and washed with TBS-T three times. Finally, the color reaction was developed with a substrate solution (100 μl 0.0015% H_2_O_2_ plus 120 μg/ml TMB [3,3',5,5'-tetramethylbenzidine]). The optical density (OD) was measured at 450 nm with subtracted the reference wavelength (OD_630_) by using a Spectra Max 190 microplate spectrophotometer (Molecular Devices) after stopping the reaction with 50 μl 3N H_2_SO_4_.

### Enzyme-linked immunosorbent assay (ELISA) and Western blotting

Reactivity of HBsAg from the transfected cell lysates and the culture supernatants was determined by using commercial ELISA kits (a Rapid II kit from Beacle Inc., Kyoto, and HBs ELISA Kit from Bioneovan Co., Ltd., Beijing) according to the manufacturer’s instructions. In Western blotting analysis, the respective protein samples prepared from the transfected cell lysates and the culture supernatants were mixed with 5X sample buffer (the final concentration was 1X) and boiled for 5 min at 100°C. The protein separated by SDS-polyacrylamide gel (4–12%) electrophoresis was then transferred to a PDVF membrane (Cat #162–0177; Bio-Rad) and blocked with 5% skim milk in TBS-T. The expressed HBsAg was detected by a mouse mAb (anti-Xpress^™^ antibody; Invitrogen) as a primary antibody followed by HRP-labeled goat polyclonal anti-mouse immunoglobulins (Dako) as a secondary antibody. Both antibodies were diluted at 1:5000 in solution 1 and solution 2 (Can Get Signal^™^; Toyobo), respectively. Chemiluminescence was visualized by Chemi-Doc (Bio-Rad).

### Immunofluorescence staining of transfected cells

The transfected cells were washed with PBS, fixed with 4% paraformaldehyde in PBS and permeabilized with 0.1% Triton X-100 in PBS. The cells were then incubated with the mouse mAb (anti-Xpress^™^ antibody; Invitrogen) diluted in PBS (1:500) containing 0.1% bovine serum albumin (BSA) and 0.02% sodium azide. After overnight incubation at 4°C, the cells were washed three times with PBS-T. Fluorescent-labeled secondary antibodies (Alexa Fluor 488, a goat anti-mouse IgG; Molecular Probes) were added to the cells, and then the cells were incubated at room temperature for at least 3 h. Thereafter, the cell nuclei were counterstained with DAPI and the slides were mounted with glycerol for confocal microscopy analysis (TCS SP8; Leica Microsystems).

### Antigenicity prediction of HBsAg

The amino acid sequences of the wt- and mt-HBsAgs were analyzed with the Jameson-Wolf algorithm by using the Protean application of Lasergene 13 (DNASTAR Inc., Madison, WI) to predict the antigenicity index according to the previously described method [44, 45]. In this algorithm-based method, an antigenic index (A*i*) is calculated using a statistical equation that combines various parameters/characteristics of primary amino acid sequences, such as hydrophobicity (Hopp-Woods), surface probability (Emini), flexibility of the protein backbone (Karplus-Schulz) and secondary structure prediction (Chou-Fasman and Garnier). Possible antigenic determinants are indicated by the positive A*i* value cluster. Thus, the specific A*i* values are generated from linear amino acid sequences that can be used to predict antigenic determinants or sites of protein, since there is a strong correlation between the predicted A*i* and known structural data.

### Software and statistical analysis

The amino acid residues of all the wt- and mt-HBsAgs were viewed and aligned using a CLC sequence viewer (http://www.clcbio.com). ExPASy (http://web.expasy.org) was used to translate nucleotides to protein sequences. Analysis of statistically significant differences in antigenicity among the wt- and mt-HBsAgs was carried out using a paired *t*-test, with values of *p*<0.05 considered to indicate significance. The results were presented as the mean ± standard error of the mean (SEM).

## Results

### HBsAg expression and secretion into the culture supernatant

**Fig 1:**
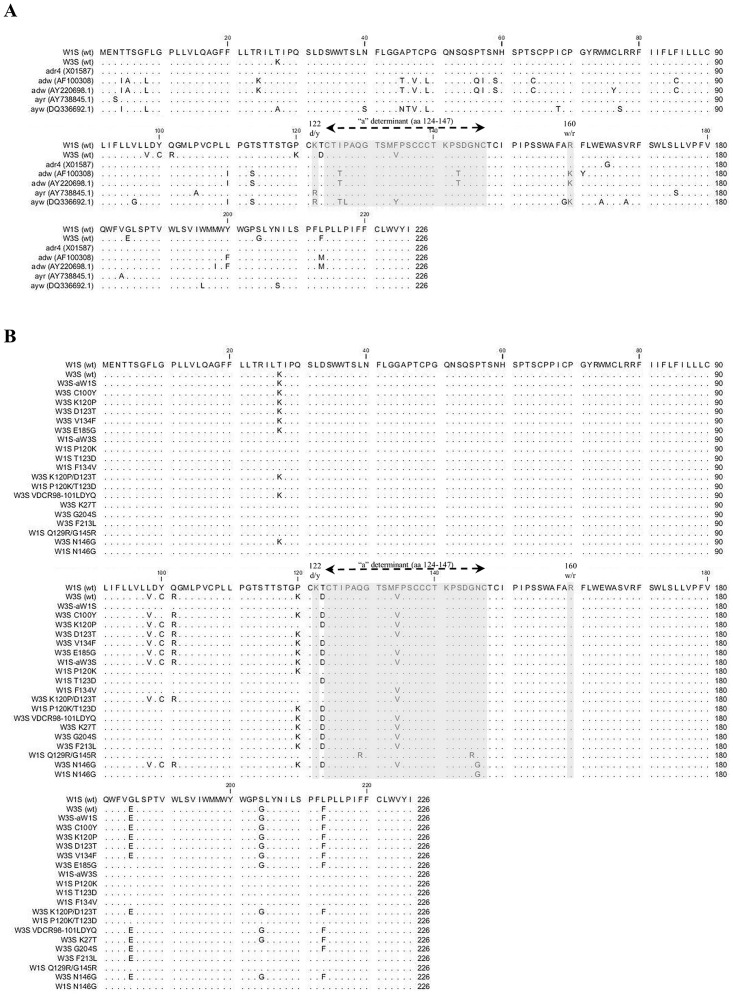
Amino acid sequence comparison of wild-type and mutated HBsAg. (A) Amino acid alignment of the wild-type HBsAgs (W1S, W3S and adr4 [X01587]) with other published sequences (adw [AF100308], adw [AY220698.1], ayr [AY38845.1] and ayw [DQ336692.1]). Only the different amino acids at the positions are represented. The dot represents the same amino acid as the first line. (B) The amino acid (aa) sequence alignment of the wild-type (W1S and W3S) and different mutants of HBsAg. The amino acid residues identical to those of HBsAg (W1S) are indicated by dots.

**Fig 2:**
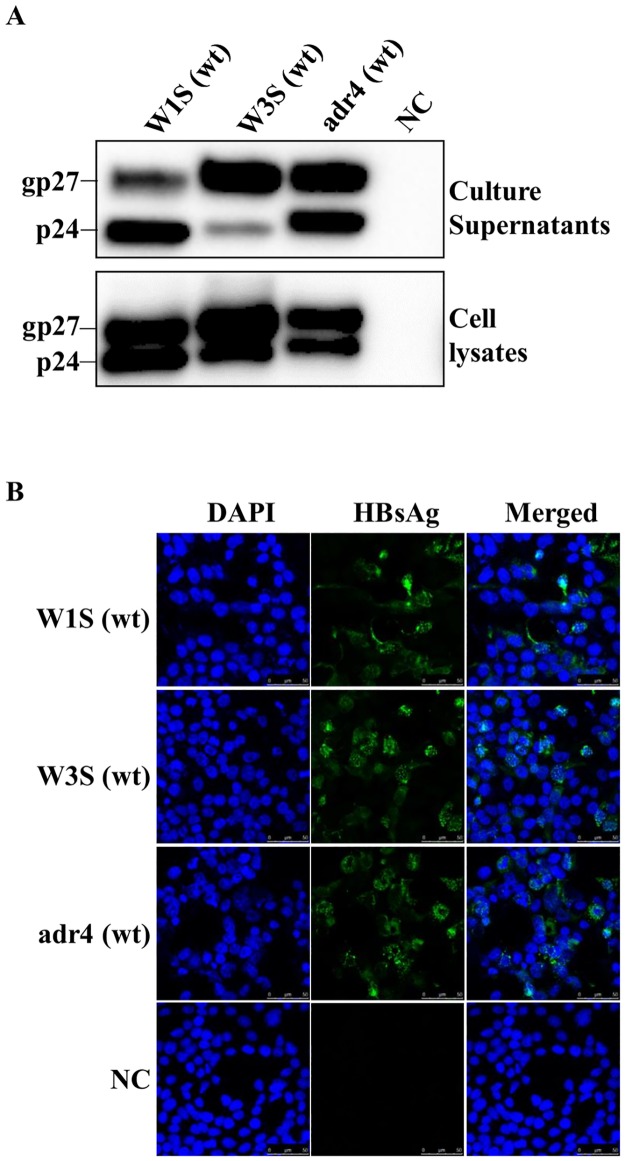
Expression of the wild-type HBsAg. (A) Western blotting analysis. HEK293-T cells were transfected with plasmid vectors expressing the wild-type HBsAgs (W1S, W3S and adr4). The expressed HBsAgs were purified from the culture supernatants and the cell lysates 72 h after transfection and an equal volume of each sample was run on SDS-PAGE. An anti-Xpress mAb against the Xpress tag was used to detect each HBsAg. (B) Immunofluorescence assay. Cells on the culture slides were fixed 72 h after transfection and stained with an anti-Xpress mAb as for the Western blot followed by an anti-mouse IgG conjugated with Alexa Fluor 488. Cell nuclei were stained with DAPI. Non-transfected cells were used as a negative control (NC). Each experiment was performed independently three times and one representative result is shown. wt: wild-type.

Three HBsAg cDNAs, namely W1S, W3S (subtype adr) and adr4 (subtype adr, genotype C), were cloned into a mammalian expression vector (pEBV His) and expressed by transient transfection in HEK293-T cells. Their amino acid sequences are shown in Fig 1A in comparison with those of the other subtypes, adw, ayr and ayw. We performed Western blotting and IFA staining to detect the expressed HBsAg using a specific antibody targeting the Xpress tag in the vector. We found that all three wt HBsAg could be detected by Western blotting from both the cell lysates and the culture supernatants (Fig 2A). Two bands of the Western blot should indicate the glycosylated and non-glycosylated isoforms of HBsAg, which were also reported previously [12, 39]. Our results showed that the glycosylated isoform of W3S HBsAg from both the supernatants and the cell lysates was more predominant in the case of W3S, compared with the cases of W1S and adr4. The dissimilarities in the HBsAg sequence might have affected the ratios of HBsAg isoforms. In addition, all the HBsAgs were positively stained by IFA using an anti-Xpress mAb and evenly distributed in the subcellular region, confirming that all the wt HBsAgs were expressed at comparable levels in the transfected cells (Fig 2B). These findings indicated that the three original HBsAgs (W1S, W3S and adr4) were well-expressed and secreted into the culture medium.

### Reactivity of HBsAg with commercial ELISA

**Fig 3:**
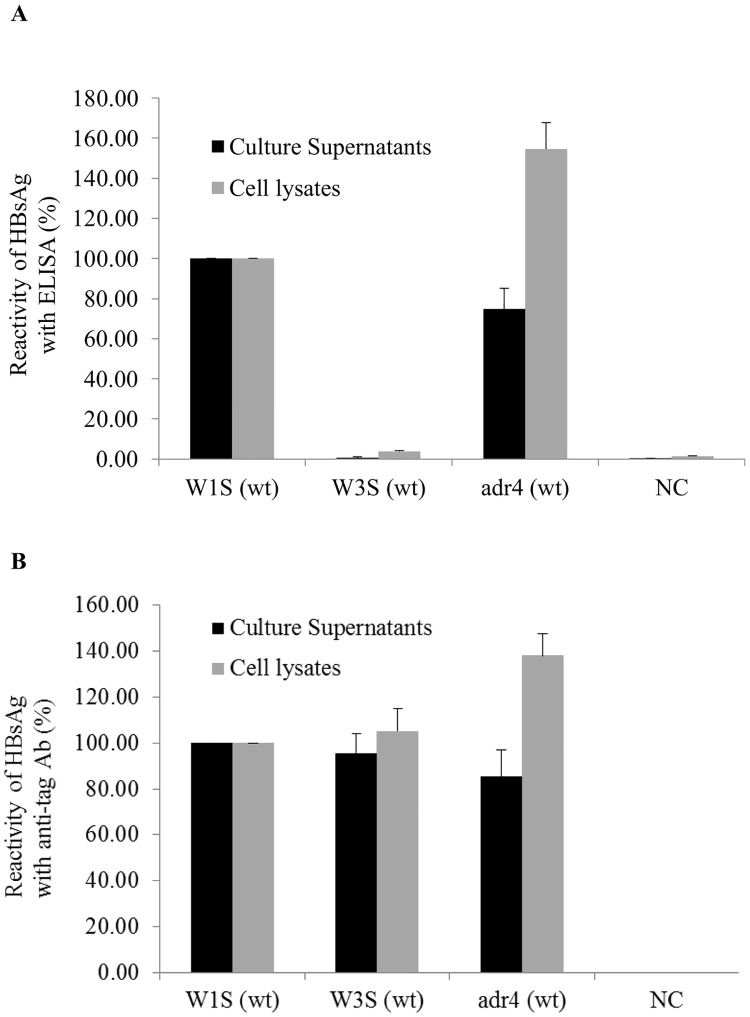
Reactivity of HBsAg (W1S, W3S and adr4) with a commercial ELISA and an anti-tag Ab. All the wild-type HBsAgs (W1S, W3S and adr4) were expressed by transient transfection in HEK 293-T cells. The culture supernatants and the cells were collected 72 h after transfection. (A) Reactivity of HBsAg from the culture supernatants and the cell lysates with a commercial ELISA kit. (B) Detection and reactivity of the wild-type HBsAg of PEG-precipitated culture supernatants and cell lysates against anti-Xpress mAb. The expressed HBsAgs were captured on a Ni-coated plate and reacted with an anti-Xpress Ab followed by anti-mouse Ab conjugated with HRP. The reactivity of HBsAgs was presented as the percentage of the OD_450-630_ of samples compared to W1S. The OD values of HBsAg ELISA and the anti-tag reactivity were normalized by the ratios of densitometric intensity of the Western blotting compared to W1S (Fig 2A). Non-transfected cells served as a negative control (NC). The mean value of three independent experiments is shown as the final reactivity of HBsAg. Error bars are shown as the standard error of means (SEM). wt: wild-type.

Mutation in the HBsAg-coding region might change the binding specificity with an anti-HBs Ab [19, 46]. We found natural mutations in the HBsAg-coding region of the W1S, W3S and adr4 by comparing them with other published sequences (Fig 1A), and the antigenicity of all three HBsAgs either from the transfected cell lysates or the culture supernatants was tested with commercially available ELISA kits. Based on the ELISA reactivity, the W1S and adr4 HBsAgs were clearly detected in both cell lysates and culture supernatants, whereas the W3S was undetectable using the same kit (Fig 3A). We do not know what kind of antibody is included in the kits, which is probably secret, but we have verified that they could detect adw (Genbank accession No; X02763), adr (X01587) and ayw (U95551) subtypes, which were cloned a long time ago, when they were expressed (data not shown in part). And they clearly detected adr4 (wt; X01587) and W1S (wt) but unable to detect W3S (wt) like as mock infection.

On the other hand, all the wt-HBsAgs (W1S, W3S and adr4) were clearly detected on a histidine binding assay plate (HIS-Select High Capacitty (HC) Nickel coated plates; Sigma-Aldrich) with the anti-Xpress mAb (Fig 3B) in addition to the reactivity against an anti-tag Ab in the Western blot (Fig 2A). Similarly, we also confirmed the results using other commercial HBsAg ELISA kits (data not shown). Thus, we speculated that the impaired reactivity (antigenicity) of the W3S HBsAg might have been due to natural mutations in its amino acid sequence that occurred at different positions compared to those in the adr4 and W1S HBsAgs (Fig 1A).

Since multiple amino acid residues within the MHR might be crucial for HBsAg antigenicity [19, 20], we focused on identifying a specific amino acid residue(s) that played an important role in the complete loss of antigenicity of W3S HBsAg. For this purpose, we constructed a series of synthetic mutants of W3S to observe their potential recovery of antigenicity; the mutants were based on the W1S amino acid sequence, because it was easily determined with ELISA kits (Fig 3A). These synthetic mutants were made by either single or multiple amino acid substitutions within or outside the “a” determinant of HBsAg (Fig 1B).

**Fig 4:**
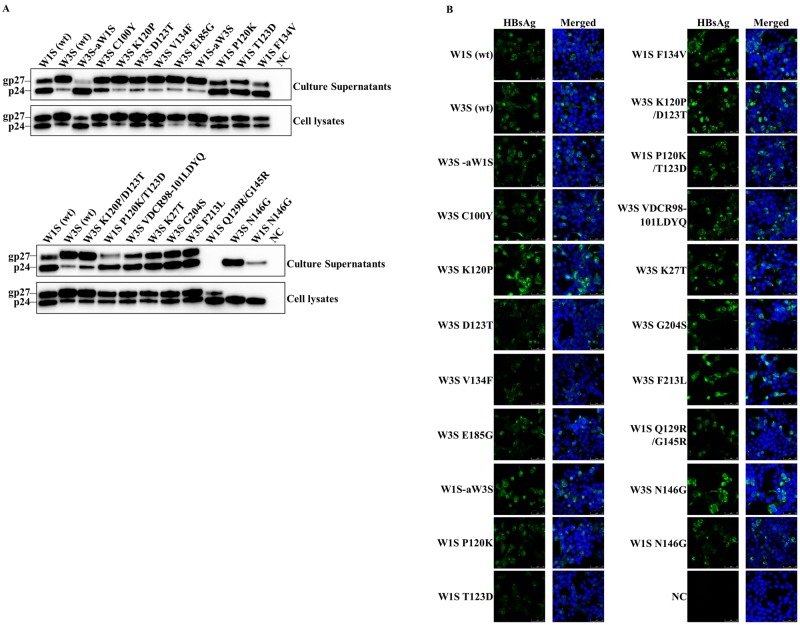
Detection of the wild-type and mutated HBsAg with Western blot and immunofluorescence analysis. (A) Western blot analysis. The HEK 293-T cells were transfected with plasmid vectors expressing the wild-type and mutants of W3S and W1S HBsAg. The expressed HBsAg was purified from the culture supernatant and the cell lysate 72 h after transfection and subjected to Western blot with an anti-Xpress mAb against the Xpress tag to detect the HBsAg. The upper panels shows blots from the culture supernatants and the lower one blots from cell lysates. (B) Immunofluorescence assay. The cells on the culture slides were fixed 72 h after transfection and stained with an anti-Xpress mAb against the Xpress tag followed by an anti-mouse IgG conjugated with Alexa Fluor 488. The cell nuclei were stained with DAPI. Non-transfected cells were used as a negative control (NC). Each experiment was performed independently three times and one representative result is shown. wt: wild-type.

**Fig 5:**
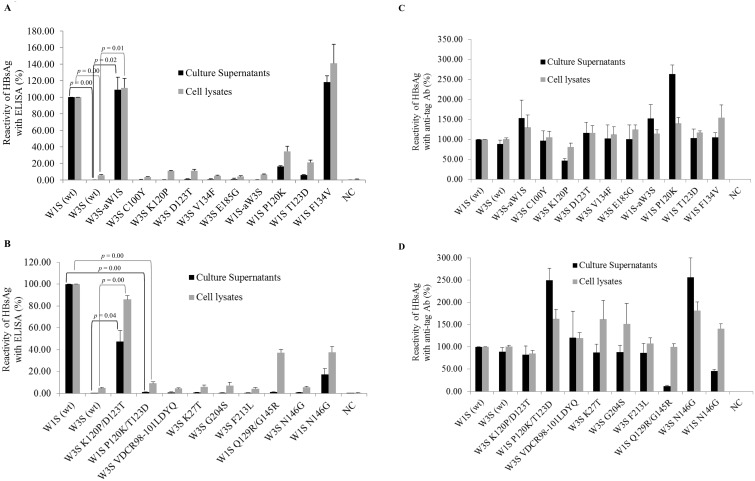
Reactivity of the wild-type HBsAg in a commercial ELISA assay and against an anti-tag mAb. All the mutants of W3S and wild-type HBsAgs (W1S and W3S) were expressed by transient transfection in HEK 293-T cells. The culture supernatants and the cells were collected 72 h after transfection. (A and B) Reactivity of the wild-type and mtHBsAg from culture supernatants and cell lysates on a commercial ELISA kit. (C and D) Detection and reactivity of the wild-type and mtHBsAg from PEG-precipitated culture supernatants and cell lysates against an anti-Xpress mAb. The reactivity of HBsAgs was presented as the percentage of the OD_450-630_ of samples compared to W1S in all cases (A, B, C and D). The OD values of HBsAg ELISA and anti-tag reactivity (except W1S N146G from the culture supernatant) were normalized by the ratios of densitometric intensity of Western blotting compared to W1S, except W1S Q129R/G145R from the supernatants, since this mutant exhibited a remarkable loss in secretion ability (Fig 4A). Non-transfected cells served as negative control (NC). The mean value of three independent experiments is shown as the final reactivity of HBsAg. Error bars are shown as the standard errors of means (SEM). wt: wild-type; mt: mutant.

We first replaced the amino acid residues of MHR of W3S with W1S (W3S-aW1S). Five amino acid mutations were found at different positions within the MHR (amino acid residues 99 to 169) of W3S, compared to W1S (Fig 1B). We also generated different single amino acid-substituted mutants of W3S within the MHR at C100Y, K120P, D123T and V134F to investigate whether their specific and individual amino acid sequences had an effect on HBsAg antigenicity (Fig 1B), and another single amino acid-substituted mutant of W3S (E185G) outside the MHR of HBsAg to check its effect on antigenicity (Fig 1B). Immunofluorescence and Western blot results demonstrated that all the above mutants of W3S were well-expressed and secreted into the culture medium (Fig 4A and 4B). When a commercial immunoassay was used to measure the antigenic reactivity, only the (W3S-aW1S) mutant exhibited an almost complete recovery of antigenicity, whereas the antigenicities of the K120P and the D123T mutants were recovered to a much smaller degree (Fig 5A). However, three other single amino acid-substituted mutants; C100Y, V134F and E185G did not show any effect on HBsAg antigenicity (Fig 5A).

To confirm the effect of amino acid mutations within the MHR, we replaced the amino acids of the MHR of the W1S HBsAg with those of the MHR of the W3S HBsAg, resulting in the mutant W1S-aW3S, and also tested the antigenicity of three single reverse mutants of W1S, i.e., P120K, T123D and F134V, to determine their effects on these specific positions. The results showed that the W1S-aW3S mutant completely lost its reactivity, while the P120K and the T123D mutants exhibited a significant reduction in reactivity in the immunoassay (Fig 5A). The mutant W1S F134V showed no change in HBsAg antigenicity. Based on the above results, we hypothesized that more than one amino acid within and/or outside the MHR, particularly at positions P120 and T123, was responsible for the antigenicity of HBsAg. Since all the mutants exhibited good reactivity, when tested with a histidine binding assay plate using the anti-Xpress mAb (Fig 5C), the HBs antigenicity was not attributed to their expression, but simply to the loss of their HBs antigenic determinants.

From the results shown above, lysine (K120) and aspartate (D123) would be expected to be very important for HBsAg antigenicity, since there was a huge reduction in W1S reactivity when the P120 and T123 were changed to K and D, respectively. Moreover, among the single mutants of W3S described above, only the W3S K120P and W3S D123T showed slight increases in the reactivity of HBsAg (Fig 5A). We therefore generated two double amino acid-substituted mutants, W3S K120P/D123T and W1S P120K/T123D, to test the simultaneous effect of amino acids at positions 120 and 123. We found that the antigenicity of the mutant W3S K120P/D123T was restored to a level nearly comparable to the antigenicity of W1S HBsAg (Fig 5B). On the other hand, W1S P120K/T123D exhibited a complete loss of the reactivity (Fig 5B).

Next, we prepared different multiple mutants of W3S along with the single one to check the combined effects of multiple amino acid substitutions on the HBsAg antigenicity. The multiple amino acid substitutions were located both within the MHR of W3S and outside of it, resulting in the mutants W3S VDCR98-101LDYQ (V98L/C100Y/R101Q), K27T (K27T/VDCR98-101LDYQ), G204S (K27T/VDCR98-101LDYQ/G204S), and F213L (K27T/VDCR98-101LDYQ/G204S/F213L) (Fig 1B). None of these multiple amino acid-substituted mutants were found to be reactive using the same commercial ELISA kit (Fig 5B). However, immunofluorescence assay and Western blotting confirmed that the levels of expression and secretion were comparable among all the double and multiple mutants (Fig 4A and 4B). Thus it was clear that the mutants were almost equally reactive against the anti-Xpress mAb, especially the mutants from the cell lysates (Fig 5C and 5D).

Among the mutants tested in this analysis, the supernatants of W1S Q129R/G145R showed remarkably reduced reactivity both in the HBsAg ELISA and against the anti-tag Ab, probably due to the secretion defect as reported previously [39].

### Effect of mutation at the N-linked glycosylation site (N146) on HBsAg antigenicity

Our Western blot analysis revealed that the level of the glycosylated isoform of the wt-W3S HBsAg was much greater than that of W1S. Interestingly, the W1S-aW3S mutant showed a higher glycosylation isoform as W3S HBsAg and vice versa and W3S-aW1S had an opposite phenotype (Fig 4A). Though we had thought that the higher glycosylation of W3S might affect the HBsAg antigenicity, the W3S N146G could not recover its antigenicity while the mutant W1S N146G showed only modestly reduced reactivity (Fig 5B). Thus, it was found that glycosylation at N146 had nothing to do with the HBsAg antigenicity.

### Changes in the predicted antigenicity index due to mutations in the HBsAg sequence

Mutations in the HBsAg sequence at specific amino acid positions may also alter the A*i* values of up and downstream amino acids [45, 47]. Therefore, we also analyzed the amino acids sequences of the wt- and mt-HBsAg by the Jameson-Wolf algorithm to check for changes in the A*i* values of specific mutations and their effect on the A*i* values of their up and downstream amino acids. Here, the A*i* of amino acid positions 110 to 160 as well as the ELISA based results showed that mutations within this region would be important for HBsAg antigenicity (Table 1). The single amino acid-substituted mutations within the first antigenic loop, W1S P120K and W1S T123D, were predicted to affect ten of their surrounding amino acids (aa 114 to 124 for P120K and aa 116 to 126 for T123D) with A*i* changes at a magnitude of -0.4 to +1.18 and -0.27 to +1.43, respectively. Double mutations at these two positions, P120 and T123 with lysine (120K) and aspartate (123D), respectively, should affect the A*i* of thirteen amino acids including their positions (aa 114 to 126) that also increased the degree of changes (-0.4 to +1.48). Substitution of glutamine at position 129 with arginine (Q129R) altered the A*i* of nine amino acids around this position with -0.4 to +1.85 magnitude, which might have led to a secretion defect of the HBs. On the other hand, the mutation at position 134 with valine (F134V) affected only six surrounding amino acids with very minor changes of A*i* (-0.4 to +0.3). Interestingly, the predicted antigenic index analysis of amino acid mutation at position 145 (G145R) altered the A*i* of seventeen amino acids with a lesser degree of changes (-0.2 to +1.4). However, this result agreed with previous findings, which showed that glycine-to-arginine mutation at this position markedly affected its downstream region [47]. Only one amino acid was affected (-0.05 to +0.15) due to the mutation at 146 in which asparagine was replaced with glycine (W1S N146G). Thus, the amino acid changes to K at P120 and to D at T123, respectively, seem to have had a serious effect on the structure of the “a” determinant.

**Table 1:** Antigenicity index (A*i*) prediction of HBsAg with Jameson-Wolf algorithm within amino acids 110–160.

HBsAg	W1S (wt)		W3S (wt)		W1S P120K		W1S T123D		W1S F134V		W1S P120K/T123D		W1S Q129R/G145R		W1S N146G	
Positions	Residues	A*i*	Residues	A*i*	Residues	A*i*	Residues	A*i*	Residues	A*i*	Residues	A*i*	Residues	A*i*	Residues	A*i*
110	Leu	-0.05	Leu	-0.05	Leu	-0.05	Leu	-0.05	Leu	-0.05	Leu	-0.05	Leu	-0.05	Leu	-0.05
111	Pro	-0.05	Pro	-0.05	Pro	-0.05	Pro	-0.05	Pro	-0.05	Pro	-0.05	Pro	-0.05	Pro	-0.05
112	Gly	0.35	Gly	0.35	Gly	0.35	Gly	0.35	Gly	0.35	Gly	0.35	Gly	0.35	Gly	0.35
113	Thr	0.4	Thr	0.4	Thr	0.4	Thr	0.4	Thr	0.4	Thr	0.4	Thr	0.4	Thr	0.4
114	Ser	0.4	Ser	***0***	Ser	***0***	Ser	0.4	Ser	0.4	Ser	***0***	Ser	0.4	Ser	0.4
115	Thr	0.4	Thr	***0***	Thr	***0***	Thr	0.4	Thr	0.4	Thr	***0***	Thr	0.4	Thr	0.4
116	Thr	0.65	Thr	***0*.*34***	Thr	***0*.*34***	Thr	***0*.*4***	Thr	0.65	Thr	***0*.*34***	Thr	0.65	Thr	0.65
117	Ser	1.3	Ser	***2*.*08***	Ser	***2*.*08***	Ser	***1*.*11***	Ser	1.3	Ser	***2*.*08***	Ser	1.3	Ser	1.3
118	Thr	1.2	Thr	***2*.*27***	Thr	***2*.*27***	Thr	***1*.*07***	Thr	1.2	Thr	***2*.*27***	Thr	1.2	Thr	1.2
119	Gly	2.05	Gly	***2*.*91***	Gly	***2*.*91***	Gly	***1*.*78***	Gly	2.05	Gly	***2*.*91***	Gly	2.05	Gly	2.05
**120**	Pro	2.5	**Lys**	**3.4**	**Lys**	**3.4**	Pro	***2*.*59***	Pro	2.5	**Lys**	**3.4**	Pro	2.5	Pro	2.5
121	Cys	2.25	Cys	***2*.*91***	Cys	***2*.*61***	Cys	***3*.*1***	Cys	2.25	Cys	***2*.*91***	Cys	2.25	Cys	2.25
122	Lys	2	Lys	***2*.*57***	Lys	***2*.*27***	Lys	***2*.*79***	Lys	2	Lys	***2*.*57***	Lys	***1*.*6***	Lys	2
**123**	Thr	0.35	**Asp**	**1.83**	Thr	***1*.*53***	**Asp**	**1.78**	Thr	0.35	**Asp**	**1.83**	Thr	0.35	Thr	0.35
124	Cys	-0.05	Cys	***1*.*04***	Cys	***0*.*44***	Cys	***1*.*32***	Cys	-0.05	Cys	***1*.*04***	Cys	-0.05	Cys	-0.05
125	Thr	-0.3	Thr	***0*.*3***	Thr	-0.3	Thr	***0*.*61***	Thr	-0.3	Thr	***0*.*3***	Thr	-0.3	Thr	-0.3
126	Ile	-0.6	Ile	***-0*.*3***	Ile	-0.6	Ile	***-0*.*3***	Ile	-0.6	Ile	***-0*.*3***	Ile	***-0*.*05***	Ile	-0.6
127	Pro	-0.6	Pro	-0.6	Pro	-0.6	Pro	-0.6	Pro	-0.6	Pro	-0.6	Pro	***0*.*6***	Pro	-0.6
128	Ala	0.35	Ala	0.35	Ala	0.35	Ala	0.35	Ala	0.35	Ala	0.35	Ala	***1*.*4***	Ala	0.35
**129**	Gln	0.8	Gln	0.8	Gln	0.8	Gln	0.8	Gln	0.8	Gln	0.8	**Arg**	**2.4**	Gln	0.8
130	Gly	0.65	Gly	0.65	Gly	0.65	Gly	0.65	Gly	0.65	Gly	0.65	Gly	***2*.*5***	Gly	0.65
131	Thr	0.35	Thr	0.35	Thr	0.35	Thr	0.35	Thr	0.35	Thr	0.35	Thr	***1*.*45***	Thr	0.35
132	Ser	0.15	Ser	***0*.*45***	Ser	0.15	Ser	0.15	Ser	***0*.*45***	Ser	0.15	Ser	***1*.*2***	Ser	0.15
133	Met	0	Met	***-0*.*1***	Met	0	Met	0	Met	***-0*.*1***	Met	0	Met	***0*.*5***	Met	0
**134**	Phe	-0.2	**Val**	-0.2	Phe	-0.2	Phe	-0.2	**Val**	**-0.2**	Phe	-0.2	Phe	***0*.*05***	Phe	-0.2
135	Pro	0.2	Pro	***-0*.*2***	Pro	0.2	Pro	0.2	Pro	***-0*.*2***	Pro	0.2	Pro	0.2	Pro	0.2
136	Ser	0.2	Ser	0.2	Ser	0.2	Ser	0.2	Ser	0.2	Ser	0.2	Ser	0.2	Ser	0.2
137	Cys	0.2	Cys	***-0*.*2***	Cys	0.2	Cys	0.2	Cys	***-0*.*2***	Cys	0.2	Cys	0.2	Cys	0.2
138	Cys	0.64	Cys	***0*.*24***	Cys	0.64	Cys	0.64	Cys	***0*.*24***	Cys	0.64	Cys	0.64	Cys	0.64
139	Cys	0.98	Cys	0.98	Cys	0.98	Cys	0.98	Cys	0.98	Cys	0.98	Cys	***1*.*18***	Cys	0.98
140	Thr	1.47	Thr	***1*.*07***	Thr	1.47	Thr	1.47	Thr	***1*.*07***	Thr	1.47	Thr	***1*.*67***	Thr	1.47
141	Lys	2.46	Lys	2.46	Lys	2.46	Lys	2.46	Lys	2.46	Lys	2.46	Lys	***3*.*06***	Lys	2.46
142	Pro	3.4	Pro	3.4	Pro	3.4	Pro	3.4	Pro	3.4	Pro	3.4	Pro	3.4	Pro	3.4
143	Ser	3.06	Ser	3.06	Ser	3.06	Ser	3.06	Ser	3.06	Ser	3.06	Ser	***2*.*86***	Ser	3.06
144	Asp	2.57	Asp	2.57	Asp	2.57	Asp	2.57	Asp	2.57	Asp	2.57	Asp	***2*.*72***	Asp	2.57
**145**	Gly	1.93	Gly	1.93	Gly	1.93	Gly	1.93	Gly	1.93	Gly	1.93	**Arg**	**2.38**	Gly	1.93
**146**	Asn	1.19	Asn	1.19	Asn	1.19	Asn	1.19	Asn	1.19	Asn	1.19	Asn	***1*.*74***	**Gly**	**1.19**
147	Cys	-0.3	Cys	-0.3	Cys	-0.3	Cys	-0.3	Cys	-0.3	Cys	-0.3	Cys	***1*.*1***	Cys	-0.3
148	Thr	-0.6	Thr	-0.6	Thr	-0.6	Thr	-0.6	Thr	-0.6	Thr	-0.6	Thr	***-0*.*3***	Thr	-0.6
149	Cys	-0.6	Cys	-0.6	Cys	-0.6	Cys	-0.6	Cys	-0.6	Cys	-0.6	Cys	-0.6	Cys	-0.6
150	Ile	-0.6	Ile	-0.6	Ile	-0.6	Ile	-0.6	Ile	-0.6	Ile	-0.6	Ile	***-0*.*53***	Ile	-0.6
151	Pro	-0.6	Pro	-0.6	Pro	-0.6	Pro	-0.6	Pro	-0.6	Pro	-0.6	Pro	***-0*.*46***	Pro	-0.6
152	Ile	-0.45	Ile	-0.45	Ile	-0.45	Ile	-0.45	Ile	-0.45	Ile	-0.45	Ile	***-0*.*24***	Ile	-0.45
153	Pro	-0.05	Pro	-0.05	Pro	-0.05	Pro	-0.05	Pro	-0.05	Pro	-0.05	Pro	***0*.*23***	Pro	***0*.*15***
154	Ser	0.35	Ser	0.35	Ser	0.35	Ser	0.35	Ser	0.35	Ser	0.35	Ser	***0*.*7***	Ser	0.35
155	Ser	0.35	Ser	0.35	Ser	0.35	Ser	0.35	Ser	0.35	Ser	0.35	Ser	***0*.*63***	Ser	0.35
156	Trp	-0.2	Trp	-0.2	Trp	-0.2	Trp	-0.2	Trp	-0.2	Trp	-0.2	Trp	***0*.*01***	Trp	-0.2
157	Ala	-0.6	Ala	-0.6	Ala	-0.6	Ala	-0.6	Ala	-0.6	Ala	-0.6	Ala	***-0*.*46***	Ala	-0.6
158	Phe	-0.6	Phe	-0.6	Phe	-0.6	Phe	-0.6	Phe	-0.6	Phe	-0.6	Phe	***-0*.*53***	Phe	-0.6
159	Ala	-0.6	Ala	-0.6	Ala	-0.6	Ala	-0.6	Ala	-0.6	Ala	-0.6	Ala	-0.6	Ala	-0.6
160	Arg	-0.6	Arg	-0.6	Arg	-0.6	Arg	-0.6	Arg	-0.6	Arg	-0.6	Arg	-0.6	Arg	-0.6

The Jameson-Wolf antigenicity index (A*i*) of HBsAs was predicted by using the Protean application of Lasergene 13 (DNASTAR Inc., Madison, WI). Mutation-induced changes in the A*i* of affected upstream and downstream amino acid residues are indicated in bold and italics.

## Discussion

More than three hundred fifty million people are infected with HBV worldwide. The detection of the hepatitis B virus surface antigen (HBsAg) is crucial for clinical diagnosis of HBV infection [13, 15, 48], and thus the emergence of antigenic escape mutants of HBsAg has had a profound effect on the vaccine design and diagnosis [20, 34, 35, 49, 50]. Although there have been a few reports analyzing mutations that affect on HBsAg antigenicity [17, 19, 39, 51], just a few comparison in the HBs 226 aa residues to develop new diagnostic assays and prophylactic measures. Here, we found that two natural amino acid mutations, lysine at 120 and aspartic acid at 123, were simultaneously responsible for the production of completely undetectable HBsAg (HBsAg with complete loss of antigenicity), when tested using commercial ELISA kits. On the other hand, these residues individually played crucial roles for the reduced reactivity of HBsAg against the immunoassays. In addition, we also showed that a mutation in the conserved N-linked glycosylation site (N146) might reduce the binding capacity of HBsAg with anti-HBsAb without leading to diagnostic failure.

*In vitro* expression and secretion of HBsAg in mammalian cells could be checked with two reliable methods, immunofluorescence assay and Western blotting analysis, as previously reported [19, 51–53]. The phenotypic characteristics and/or antigenic reactivity of the wt- and mt-HBsAg against anti-HBs antibodies are usually analyzed by commercial immunoassays [17, 39, 52, 54].

Several epidemiological studies have suggested that single and/or multiple amino acid mutations were very frequent in the HBsAg sequences, especially in the MHR, clinically isolated from HBV-infected patients [55–57]. In addition, mutations in the MHR might escape antibody neutralization and host immune response [20]. HBsAg antigenicity was analyzed by dividing the MHR (amino acids 99 to 169) into subregions, and no antigenic determinants were found within the region from amino acids 99 to 119 (MHR 1) [11, 17]. Accordingly, we did not find any antigenic or secretory defect arising from the mutation at amino acid position 100 (Y100C), either when present singly (C100Y) or together with other mutations (VDCR98-101LDYQ), although Motta-Castro et al. observed a low level of HBV load in the serum with a C100Y mutation of the surface gene [58]. However, Ireland et al. found no reactivity of HBsAg using two commercial diagnostic assays whose sequences had simultaneous mutations at various positions, including an amino acid at 100 (Y100S), and this may have been due to mutations at other sites (T118V/R122K/M133I/Y134N/P142S/S143L/G145K) [59]. They also found reduced reactivity of HBsAg mutated at two positions simultaneously (Y100C/P120T).

Several reports have described antigenicity analyses of mutated HBsAgs that were mainly focused on the amino acid residues within the antigenic loops of the “a” determinant [17, 19, 39, 51, 59]. HBsAgs from OBI patients with mutation at amino acids positions 124, 141 and 144 showed reduced sensitivity when analyzed with different commercial immunoassays [19]. Natural mutation at the residue position 120 is very common, and has also been identified in patients with OBI, but the replacement of proline at this position (P120) with threonine (P120T) has been reported to have no effect on HBsAg antigenicity [17, 19]. On the other hand, when threonine at position 123 (T123) was substituted with asparagine (T123N), the reactivity of HBsAg in the supernatant of transfected cells was significantly reduced, but HBsAg retained some degree of its reactivity with the cell lysate or with other antibodies [17, 51]. However, these studies suggested that amino acid residues at positions 120 to 123 might be crucial for HBsAg antigenicity, and the residue at position 123 might have an effect on secretion deficiency [17].

Consistent with previous reports, we also found significantly reduced antigenic reactivity of HBsAg in commercial immunoassays when independent mutation was made at position 120 with lysine (P120K) and at position 123 with aspartate (T123D). Surprisingly, we found that HBsAg lost antigenic reactivity when amino acid substitutions P120 and T123 were present simultaneously (P120K/T123D). Similarly, the antigenic sensitivity was fully recovered when we substituted the amino acid residues of these positions (120 and 123) with proline and threonine (K120P/D123T) and performed analyses using the same commercial immunoassay. The predicted secondary structure of HBsAg suggested that the amino acid residues at 120 and 123 were in the first N-terminus antigenic loop within the MHR [48, 60]. Huang et al. reported that their generated mAbs recognized this first loop (aa 113–127) and the binding was dramatically decreased by mutation in the same loop [19].

In addition, a mutation at T123 might alter the conformation and the antigenicity of HBsAg as judged by the Jameson-Wolf antigenicity index. Antigenic index (A*i*) values of four or more amino acids around amino acid position 123 were altered by a magnitude of -0.4 to +0.2 or more by the T123A or T123N mutations, respectively [45]. In this study, we found that seven up and three downstream amino acids were affected due to the mutation of threonine at position 123 by aspartate (T123D), with significant alteration of A*i* values, and A*i* degrees were notably changed from -0.4 to +1.48 due to simultaneous replacement of the amino acids at 120 and 123 with lysine and aspartate, respectively. These A*i* alterations were also strongly correlated with the antigenic reactivity of HBsAg determined by ELISA.

The biochemical properties of amino acids such as hydrophobicity and electronic charges could lead to conformational change in the HBs structure involved in the reduced HBsAg secretion and impaired reactivity with anti-HBsAgs antibodies [39]. Lysine is a positively charged amino acid and aspartate is negatively charged, whereas threonine has a neutral side chain and proline is a specially structured amino acid and the anionic carboxylate (RCOO^-^) of aspartic acid or glutamic acid and cationic ammonium (RNH_3_^+^) of lysine or the guanidinium (RNHC [NH_2_]_2_^+^) of arginine should create a salt bridge.

**Fig 6:**
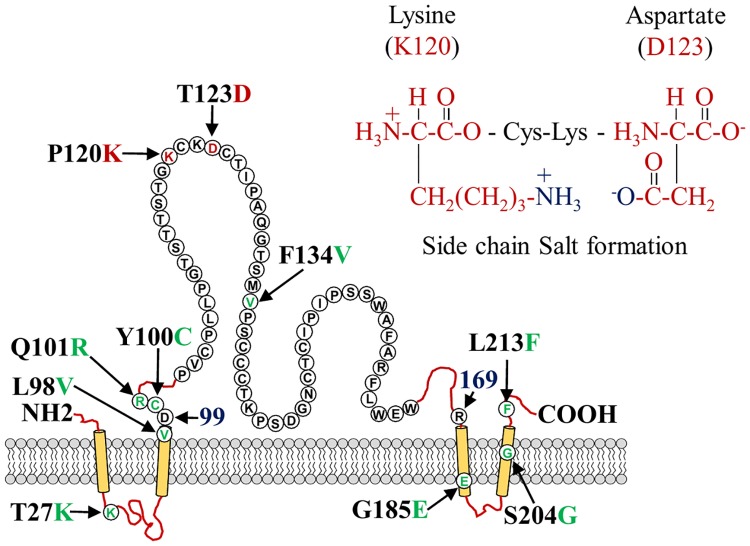
Hypothesis of the mechanism of HBV diagnostic- and immune-escape induced by the HBsAg amino acid mutations. The lysine and aspartate might form a salt bridge with their side chains and thereby change the conformation of HBsAg, leading to a loss of the binding capacity with anti-HBs Abs.

Thus, we speculated that lysine (K120) formed a salt bridge of its cationic ammonium (RNH_3_^+^) with the anionic carboxylate (RCOO^-^) of aspartic acid (D123), which led to a complete conformational alteration of HBsAg and thereby changed the antigenicity (Fig 6). It might be suggested that the proline/lysine at amino acid position 120 and threonine/aspartate at 123 simultaneously determined the antigenic phenotype of HBsAg.

An amino acid mutation was also frequently found at position 134, as detected in the HBsAg sequence isolated from a chronically HBV-infected patient with liver cirrhosis [47, 61]. Interestingly, the wt-HBs sequence (F134) was mutated to F134Y after orthopedic liver transplantation (OLT) or mutants I134F or Y134F were reverted to the wt (F134) after OLT and the predicted antigenic index was made slightly more positive due to amino acid substitution at this position either by tyrosine or isoleucine [47]. Accordingly, the antigenic reactivity against a commercial HBsAg immunoassay was increased to some extent when phenylalanine at amino acid position 134 was substituted with valine (F134V) and it was not found to be responsible for the undetectability of HBsAg.

Most of the mutations found in the upstream and downstream region of the HBsAg “a” determinant were responsible for vaccine breakthroughs that tended to cluster within known Th and/or CTL epitopes [62]. In addition, several groups predicted the secondary structure of HBsAg with at least four different transmembrane domains, but the amino acid positions and the numbers within these domains were flexible [11, 53, 60, 63]. According to the transmembrane domain structure, amino acid residues T27, G185 and S204 were thought to be buried in the transmembrane domains or in the cytosolic loop. Perhaps, due to the above, mutations found at these positions had no effect on the reactivity of HBsAg in commercial diagnostic assays (Fig 5A and 5B). Amino acid residues 206–215 of HBsAg contained an antigenic epitope specific for MHC class I T- cells (CD8) and vaccine-induced mutations such as 213M/F/I have been found within this region [62, 64]. L213 was found in the endoplasmic lumen adjacent to the C-terminus of the HBs amino acid sequence according to the predicted secondary structure. However, we found no effect of L213F mutation on the detection and the production by transient expression.

Glycine-to-arginine mutation at 145 (G145R) is a well-known mutation of HBsAg that is responsible for both immune escape and a reduction of the binding capacity in commercial diagnostic assays [39, 65, 66]. G145R was also detected in patients with orthopedic liver transplantation even after having regular hepatitis B immune globulin prophylaxis [47]. G145R alone or in combination with others mutation in the HBs sequence may also affect the antigenic structure since it is located in the antigenic loop of “a” determinant. Substitution of glutamine to arginine at position 129 (Q129R) was identified from the clinically HBV infected patients that also lied within the antigenic loop of “a” determinant. Glycine-to-alanine at 145 (G145A) and glutamine-to-arginine at 129 (Q129R) mutations were also found in the HBs amino acids sequence isolated from separate infants who already received recombinant hepatitis B vaccine [67]. Our synthetic mutant with substitutions of glycine-to-arginine at position 145 and glutamine-to-arginine at 129 (Q129R/G145R) showed significantly reduced binding efficiency especially in the supernatants (Fig 5B and 5D). In addition, the combined mutations at these two positions with arginine (Q129R/G145R) affected twenty-eight amino acids surrounding the positions with significant degrees of A*i* alteration. Therefore, this double amino acid-substituted mutation might induce a greater inhibition of the efficiency of HBsAg secretion into the extracellular space and a greater reduction of antigenic reactivity compared to either of the individual mutations singly (Q129R or G145R), suggesting the importance of these positions [19].

Moreover, a previous report revealed that some amino acid substitutions facilitated the glycosylation of HBsAg [51]. Modification of the glycosylation pattern would alter the confirmation of HBsAg, since W3S showed more glycosylation than W1S (Fig 4A), which might be associated with immune escape [12, 68]. In addition, Julithe et al. showed that the coexistence of non-glycosylated and glycosylated isoforms of HBsAg at N146 played roles in both infectivity and immune escape [68]. Our analysis of the effects of the glycosylated and non-glycosylated isoforms on HBsAg antigenicity and immunogenicity showed that mutation in the glycosylation site (N146) to glycine did not lead to diagnostic failure and only modestly reduced the HBsAg antigenic reactivity in the commercial immunoassays.

Taken together, these results demonstrate that all the wt and synthetic mutants were well expressed and secreted into the culture medium except the mutant W1S Q129R/G145R, which had a secretion deficiency. Importantly, the simultaneous effect of mutation to lysine at amino acid position 120K and mutation to aspartate at position 123D led to a critical diagnostic failure of HBV infection by the commercial HBsAg immunoassays, though single mutations at these positions (either 120K or 123D) were separately responsible for only a partial reduction of the binding capacity of HBsAg against anti-HBs.
